# Evaluating human cancer cell metastasis in zebrafish

**DOI:** 10.1186/1471-2407-13-453

**Published:** 2013-10-04

**Authors:** Yong Teng, Xiayang Xie, Steven Walker, David T White, Jeff S Mumm, John K Cowell

**Affiliations:** 1 Cancer Center, Georgia Regents University, Augusta, GA, USA; 2 Department of Cellular Biology and Anatomy, Georgia Regents University, Augusta, GA, USA; 3 Vision Discovery Institute, Georgia Regents University, 1120 15th Street, Augusta, GA 30912, USA

**Keywords:** Cancer cells, Zebrafish, Metastasis, Invasion, Mouse

## Abstract

**Background:**

*In vivo* metastasis assays have traditionally been performed in mice, but the process is inefficient and costly. However, since zebrafish do not develop an adaptive immune system until 14 days post-fertilization, human cancer cells can survive and metastasize when transplanted into zebrafish larvae. Despite isolated reports, there has been no systematic evaluation of the robustness of this system to date.

**Methods:**

Individual cell lines were stained with CM-Dil and injected into the perivitelline space of 2-day old zebrafish larvae. After 2-4 days fish were imaged using confocal microscopy and the number of metastatic cells was determined using Fiji software.

**Results:**

To determine whether zebrafish can faithfully report metastatic potential in human cancer cells, we injected a series of cells with different metastatic potential into the perivitelline space of 2 day old embryos. Using cells from breast, prostate, colon and pancreas we demonstrated that the degree of cell metastasis in fish is proportional to their invasion potential *in vitro*. Highly metastatic cells such as MDA231, DU145, SW620 and ASPC-1 are seen in the vasculature and throughout the body of the fish after only 24–48 hours. Importantly, cells that are not invasive *in vitro* such as T47D, LNCaP and HT29 do not metastasize in fish. Inactivation of JAK1/2 in fibrosarcoma cells leads to loss of invasion *in vitro* and metastasis *in vivo*, and in zebrafish these cells show limited spread throughout the zebrafish body compared with the highly metastatic parental cells. Further, knockdown of WASF3 in DU145 cells which leads to loss of invasion *in vitro* and metastasis *in vivo* also results in suppression of metastasis in zebrafish. In a cancer progression model involving normal MCF10A breast epithelial cells, the degree of invasion/metastasis in vitro and in mice is mirrored in zebrafish. Using a modified version of Fiji software, it is possible to quantify individual metastatic cells in the transparent larvae to correlate with invasion potential. We also demonstrate, using lung cancers, that the zebrafish model can evaluate the metastatic ability of cancer cells isolated from primary tumors.

**Conclusions:**

The zebrafish model described here offers a rapid, robust, and inexpensive means of evaluating the metastatic potential of human cancer cells. Using this model it is possible to critically evaluate whether genetic manipulation of signaling pathways affects metastasis and whether primary tumors contain metastatic cells.

## Background

Metastasis is the primary cause of human cancer mortality, accounting for >90% of deaths due to cancer [1]. There is now abundant evidence that, independent of the process of cellular transformation, the metastasis phenotype is genetically controlled [2]. Metastasis is a multistep process that involves local tumor invasion followed by dissemination to, and re-establishment at, distant sites. Families of genes have been described which have no effect on cell proliferation but which can suppress or promote metastasis [3,4]. Thus, targeting metastasis may prove to be effective in reducing cancer mortality if specific targets can be identified that suppress this phenotype. Here, we present a robust *in vivo* system for rapidly and accurately evaluating the effectiveness of candidate suppressor molecules.

Much of the analysis of metastasis pathways is conducted in tightly controlled *in vitro* cell systems, usually involving overexpression or ablation of a particular gene. Assays such as wound healing, transwell motility, invasion assays and hanging drop assays have been developed which provide readouts of cellular phenotypes related to metastasis [5-7]. These assays, however, do not address the issue of intravasation of tumor cells into blood vessels and extravasation into distant organs, a process requiring an *in vivo* assay system. Typically, such assays are performed in mice using experimental or spontaneous metastasis models [8,9]. While it is ultimately necessary to demonstrate that a pathway identified *in vitro* also affects invasion and metastasis *in vivo*, mouse models have significant drawbacks: 1) it is difficult to study early stages of the process where it is necessary to rapidly evaluate whether a particular drug or genetic manipulation has affected the metastasis phenotype, 2) evaluating the complete process in mice can require up to 6 months (depending on the cell system), 3) these experiments are expensive, immunosuppressed mice are required to study human cells and per diem charges in barrier facilities are costly, 4) *in vivo* imaging of small metastatic lesions is not possible in the deep tissues of the mouse, thus typically requiring termination and autopsy, thus extrapolation across experimental populations to realize the result, 5) popular immunosuppressed mice such as, nude (nu/nu), the severe combined immunodeficiency (SCID), or mice null for the recombination activating gene (Rag), have residual immune competence, which can actually prevent metastasis and, 6) the cohort size in these experiments is often pragmatically limited by high costs, thus statistical verification of metastasis modulation cannot be adequately assessed when the effect is mild.

Zebrafish provide an experimentally and genetically tractable animal model of a wide variety of human diseases [10]. Recent studies have demonstrated that zebrafish form spontaneous tumors with similar histopathological and gene expression profiles as human tumors [11-13]. The zebrafish-cancer model overcomes the drawbacks of murine xenograft models and offers alternative options for studying human tumor angiogenesis and metastasis [14-21]. Following early reports of the application of zebrafish to evaluate metastasis [22], we now tested whether metastasis in fish faithfully reports the metastatic potential of a broad range of cancer cells. To do so, we correlated *in vitro* invasion efficacy to *in vivo* metastasis metrics following manipulation of the metastatic phenotype. Without exception, we show that gene manipulations that affect *in vitro* invasion, alter metastasis in fish in a corresponding manner, demonstrating that the zebrafish is a tractable model to assay metastatic potential of human cancer cells. We also show that primary human cancer cells can metastasize in fish and that this ability can be used to predict metastatic potential in a clinical setting.

## Results

### The endogenous metastasis phenotype of human cancer cells is maintained in zebrafish

**Figure 1:**
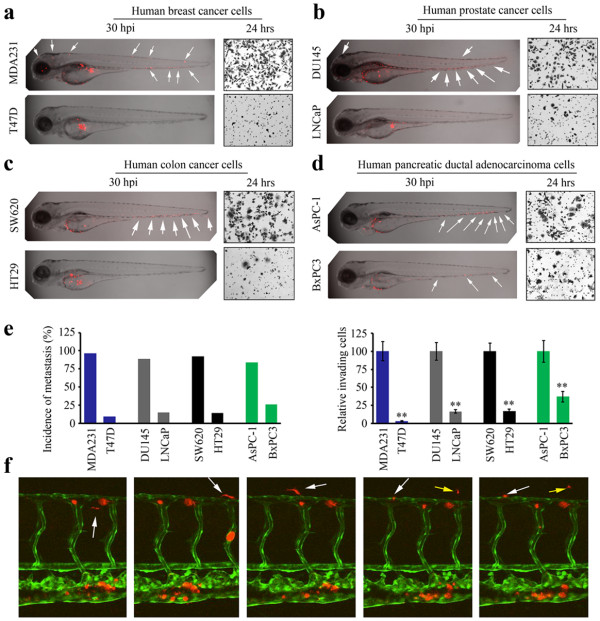
**Zebrafish faithfully report metastasis in human cancer cells.** Confocal imaging (shown as z-stack images using 40 × magnification) shows that **(a)** metastatic MDA-MB-231 (MDA231) breast cancer cells (above) are dispersed throughout the fish body (arrows), whereas non-invasive T47D cells (below) remain in the confines of the yolk sac. These results are mirrored by the Transwell invasion assays, shown in each case to the right. Similarly **(b)** invasive DU145 prostate cancer cells show extensive spread throughout the fish compared with non-invasive LNCaP cells which do not. In colon cancer cells **(c)**, highly invasive SW620 cells metastasize extensively compared with non-invasive HT29 cells. Relative invasion potential of pancreatic cancer cells **(d)** shows that more cells are distributed throughout the fish body for the relatively more invasive AsPC-1 cells compared with less invasive BxPC3 cells. Assessment of fish showing metastasis (see text) in each experiment compared with the invasion potential is shown in **(e)** showing the close correlation between relative invasion *in vitro* using the transwell assay and *in vivo* metastasis in fish. ** P > 0.001; * P >0.05. Using the *Tg(kdrl:EGFP)* transgenic zebrafish **(f)**, cancer cells (red) that have spread throughout the vasculature (green) can be seen for MDA231 as show by successive imaging of 4 hours (200 × magnification). The presence of MDA231 cells that have extravasated into the body of the fish can clearly be seen (the white arrow tracks movement of a single cell and yellow arrow indicates movement of another).

We first investigated whether human cancer cells, with known invasion/metastasis potential, could disseminate throughout the zebrafish body. To minimize the possibility that cells were introduced directly into the vasculature in error during the injection process, the fish were examined after 12 hours and those showing cells already in the vasculature were removed from further analysis. MDA-MB-231 breast cancer cells, for example, are highly invasive *in vitro* and metastasize in experimental and spontaneous murine models *in vivo*[8]. CM-Dil stained MDA-MB-231 cells were injected into the perivitelline cavity of 48 hours post fertilization (hpf) embryos and analyzed at 30 hours post injection (hpi) using confocal microscopy, where the spread of cancer cells could be seen throughout the body of the fish. In contrast, the T47D breast cancer cells, which do not typically invade or metastasize [7], did not show evidence of metastasis in the fish (Figure 1a). In parallel, the invasion potential of these cell lines was assessed *in vitro* using standard transwell assays, which was concordant with their ability to metastasize in the fish (Figure 1a). During our analysis of non-metastatic cell lines (e.g. T47D and (see below) HT29), we noted that, although the majority of fish did not show any disseminated cells within the fish body after the first 48 hour period, there were infrequent cases where cells were detected in the body of the fish. Quantitation of these cells revealed that in these cases there were never more than 5 cells outside the yolk sac region. We therefore set the cut off of >5 cells to indicate the definition of metastasis. Where dissemination was seen for the highly metastatic cells, however, there were significantly more than 5 cells within the fish body.

We have also shown previously that DU145 prostate cancer cells invade *in vitro* and metastasize *in vivo* in mouse models [9] compared with LNCaP cells which do not. As shown in Figure 1b, DU145 cells metastasize throughout the body of the fish at 30 hpi but LNCaP cells do not. The same correlation was seen for invasive SW620 and non-invasive HT29 colon cancer cells (Figure 1c), where again the correlation between invasion and metastasis in the fish was observed. Finally we studied the pancreatic ductal adenocarcinoma cells AsPC-1 and BxPC3 (Figure 1d). In this case, the AsPC-1 cells showed higher invasion potential than BxPC3 in the fish model which was consistent with *in vitro* invasion assays (Figure 1d). The extent of metastasis in fish for the highly invasive cells lines was readily apparent with large numbers of cells throughout the body of the fish (>25) after 24–48 hours. In contrast, the cells with low metastatic potential rarely showed cells in the body of the fish. These correlations were consistent within the cohort of fish used for each experiment. To evaluate the metastatic potential, therefore, we determined the number of fish that showed metastasis in the four different cancer cell systems, compared with the number that did not. In the highly invasive cell lines, MDA231 (n = 58), DU145 (n = 72), SW620 (n = 63) and ASPC-1 (n = 57), metastasis was seen in 97%, 89%, 92% and 84% of fish respectively (Figure 1e). In contrast, in the poorly invasive cells, T47D (n = 64), LNCaP (n = 53), HT29 (n = 84) and BxPC3 (n = 42) metastasis was seen in only 9%, 15%, 14% and 26% of fish respectively (Figure 1e). We then performed quantitative invasion assays (see Methods) for all of these cell lines (Figure 1e) where the relative proportion of invading cells mirrored the distribution of metastasis in the in vivo fish model.

To demonstrate that cells from the perivitelline cavity could intravasate into the circulation and extravasate into the fish body, we used the *Tg(kdrl:EGFP)* transgenic zebrafish which highlights the vasculature (Figure 1f). In this study, using invasive MDA231 cells, for example, we could clearly see CM-Dil-labeled human cancer cells both in the vasculature and in the body of the fish adjacent to the vasculature (Figure 1f). This analysis demonstrated that the human cancer cells showed the range of phenotypes associated with metastasis. In contrast, the non-invasive T47D cells, were never seen in the vasculature of the host fish, nor in tissues distant from the injection site (Figure 1a). Thus, these data suggest that zebrafish can robustly report metastasis potential of different types of human cancer cells.

### Assessment of the metastatic potential of primary human cancer cells in zebrafish

**Figure 2:**
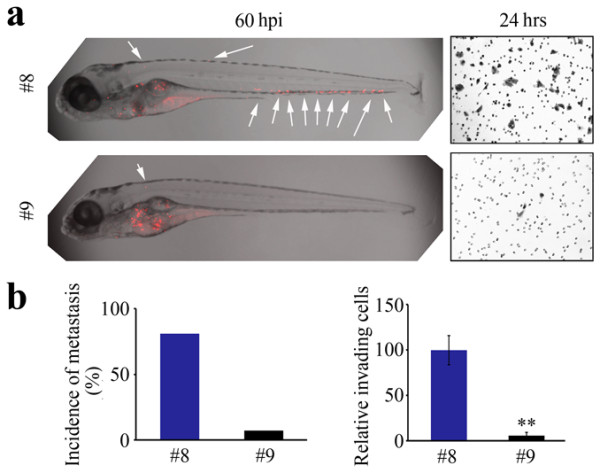
**Metastasis of primary human lung tumor cells in zebrafish.** Cells derived from short term culture of a primary human lung tumor with an undifferentiated phenotype (#8) show extensive spread throughout the body of the fish (**a**, left) and invasion *in vitro* (**a**, right). In contrast, cells from a well-differentiated adenocarcinoma of the lung (#9) show only minimal dissemination in the fish and limited invasion *in vitro*. From cohort studies **(b)**, the number of fish showing metastasis is significantly greater in #8 (shown as z-stack images using 40 × magnification). This relationship is maintained in *in vitro* invasion assays (**b**, right).

In the experiments described above, we have demonstrated that cancer cells with different *in vitro* invasion phenotypes show a parallel metastasis phenotype in zerbafish in all cases examined. These observations raised the issue whether metastatic potential in primary human cancer cells could also be assessed in this system. In a pilot proof-of-principle study, therefore, we prepared primary cultures of human lung cancer cells and maintained them for 10–15 days to collect sufficient cells for injection. Cells from only two different primary cultures were available to us and these were injected into zebrafish as described above and at 60 hpi tumor cell spread was assessed. The cells from tumor #8 showed a clear dissemination in the fish body, comparable with other highly invasive cell lines described above (Figure 2a). On the other hand, the cells from tumor #9 did not show any dissemination (Figure 2a). Histopathology of the primary tumors in these two cases, as part of routine diagnosis, showed that tumor #8 was derived from an undifferentiated squamous cell carcinoma, whereas tumor #9 was a well-differentiated adenocarcinoma. Quantitation of metastasis as defined above showed 81% (n = 42) of fish showed metastasis for cells from tumor #8 compared with only 7% (n = 57) for cells from tumor #9 (Figure 2b). Transwell invasion assays for cells from these primary cultures demonstrated that tumor #8 was highly invasive but that tumor #9 was not (Figure 2b). Thus, the relationship between invasion *in vitro* and metastasis *in vivo* in zebrafish is maintained in this analysis. Taken together, our data suggest that zebrafish may be a reliable *in vivo* model for assessment of the metastatic potential of primary human cancer cells.

### The genetic regulation of tumor metastasis is maintained in zebrafish

**Figure 3:**
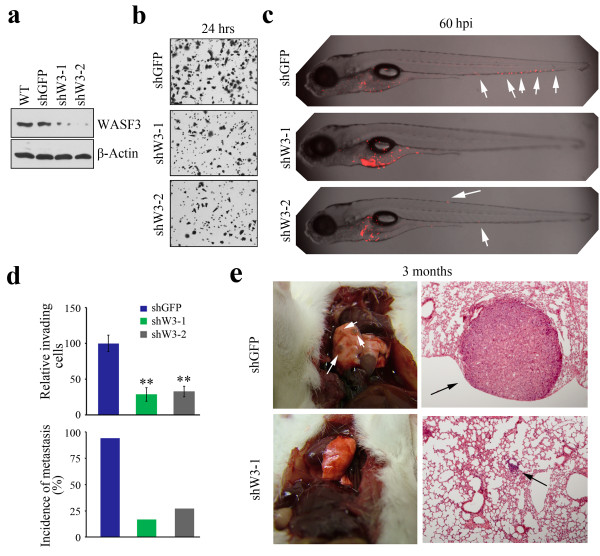
**Comparable WASF3-induced metastasis of DU145 cells in mice and fish.** Knockdown of WASF3 in DU145 cells using two different shRNA constructs (shW3-1 and shW3-2) shows significant reduction in protein levels **(a)**. DU145 cells transfected with a control shRNA (shGFP) show high level invasion *in vitro***(b)** but knockdown of WASF3 in these cells using two different shRNAs (shW3-1 and shW3-2) leads to significantly reduced invasion. In fish **(c)**, no, or reduced numbers, of WASF3 knockdown cells (shW3-1 and shW3-2) spread throughout the body of the fish, whereas the control cells (shGFP) spread extensively (arrows) (shown as z-stack images using 40 × magnification). Quantitation of invasion potential shows a significant reduction (** p >0.001) in the WASF3 knockdown cells compared with control shGFP cells (**d**, above). This reduced invasion is mirrored by the metastasis efficiency in cohort studies of fish (**d**, below). In mice **(e)**, tumor nodules (arrows) on the surface of the lungs can be seen in the control shRNA treated cells compared with cells expressing shW3-1 (left) 3 months after tail veil injection. Histological examination of the lungs of these mice shows large tumor foci from the control cells (arrows) compared with small tumor foci for the WASF3 knockdown cells.

WASF3 (or WAVE3) is a member of the Wiskott-Aldrich syndrome protein family. The structural motifs in the WASF3 protein predict that it orchestrates the reorganization of the actin cytoskeleton, which leads to the development of invadapodia and lamellipodia which facilitate invasion [4,23]. We have previously shown that WASF3 is high expressed in primary human cancer and fundamentally important for metastatic spread of cancer cells [7-9,24,25]. To relate metastasis in fish with metastasis in mice, we analyzed DU145 cells in both models. As shown in Figure 3a-b, the control DU145 cells (shGFP) which have high WASF3 expression levels were highly invasive *in vitro.* Consistently, the control DU145 cells (shGFP) showed metastasis in the fish model at 60 hpi, whereas the stable WASF3-deficient cells (shW3-1 and shW3-2) show significantly reduced metastasis (Figure 3c). Quantitation of metastasis as defined above showed 94% (n = 51) of fish showed metastasis for control cells (shGFP) compared with only 12% (n = 72, shW3-1) and 18% (n = 66, shW3-2) for WASF3 knockdown cells (Figure 3d). In a mouse model of metastasis (Figure 3e), lung surface tumors and large tumor foci throughout the lungs were seen after 3 months, while the WASF3 knockdown cells clearly showed a significant reduction in both invasion potential or metastasis capability (no detectable tumors on the lung surface and very small tumor foci throughout the lung). Thus, assays evaluating metastatic potential of cells are consistent between mouse and zebrafish.

### Quantitation of metastasis in zebrafish using Fiji algorithms

**Figure 4:**
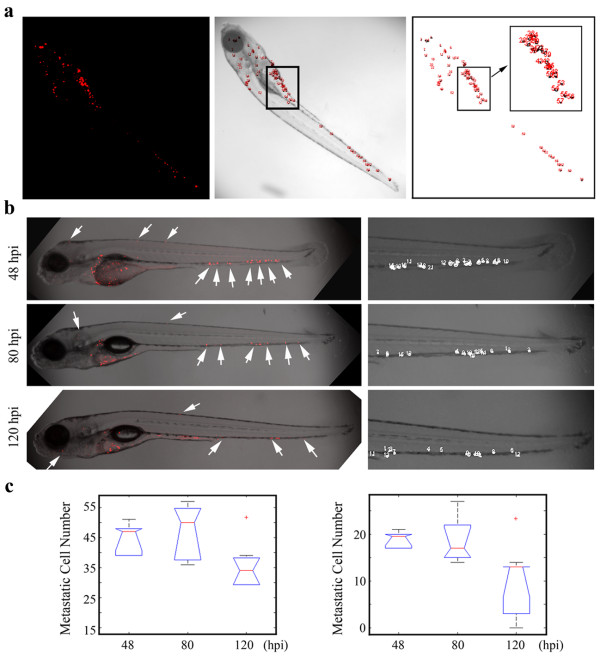
**Quantitation of metastatic cells in zebrafish using Fiji.** Fluorescent (**a**, left) and bright field (**a**, center) images of Dil-stained (red) human MDA231 cancer cells are captured by confocal microscopy. Individual red cells are counted relative to their location throughout the fish (a, center) using Fiji. Cells within the yolk sac (boxed) can be eliminated from the cell count (**a**, right). Cells were counted using this approach **(b)** either throughout the body of the fish (left) or confined to the tail region (right) in the same fish over a 48–120 hours period after injection. Cells visualized by the fluorescent dye (arrows) and following Fiji analysis (right) were plotted from the same 10 different fish at the three time points showing **(c)** no significant difference in mean number of cells counted in the whole fish body (left) up to 80 hours. Although the number of metastatic cells was reduced when cells restricted to the tail region were counted (right), there was again no significant difference in metastatic cells up to 80 hours after injection. At later stages (120 hpi), an ~25% reduction in metastatic cell numbers was observed in both the fish body and the tail alone. Data are presented as the mean of three independent experiments (n = 3) ± SEM, P < 0.05.

The transparent zebrafish embryos make imaging metastatic cells relatively straightforward using both conventional and confocal microscopy. Fish can be lightly anesthetized and photographed allowing repeat analysis of the same fish after different time intervals. Although the experiments described above show a clear distinction between the ability to metastasize and not, in certain circumstance it may be necessary to quantify the metastatic potential. To achieve this is a realistic time frame, we have modified the open source Fiji software [26] to quantitate the number of cells that have left the perivitelline space as a measure of metastasis. The photographs of the fish are used in this analysis and an example is shown in Figure 4a. In this system, the CM-Dil stained cells are imaged and counted throughout the fish. The cells remaining in the yolk sac are masked off and eliminated, and the number of cells in the fish body used as an estimate of the metastatic capability of the cells. In the example shown in Figure 4, MDA231 cells were imaged at 48 hpi in 10 different fish. The same fish were then imaged at 80 hpi (Figure 4b), where there was no significant difference in the number of metastatic cells (Figure 4c, left). At 120 hpi, however, there was a significant decline in the number of cells (Figure 4b-c). To streamline the study, we repeated the analysis where we counted only cells in the tail region, distal to the yolk sac (Figure 4b, right). Again, we saw no difference in the metastatic spread of the cells during early stages but saw a reduced number of cells at 120 hpi (Figure 4c, right). This analysis has advantages in that there is no need to assess the number of cells that remained in the yolk sac region or that had metastasized to the head region where the large area occupied by the eye presents challenges in quantitation.

### Zebrafish show variation in metastasis potential related to tumor cell progression

**Figure 5:**
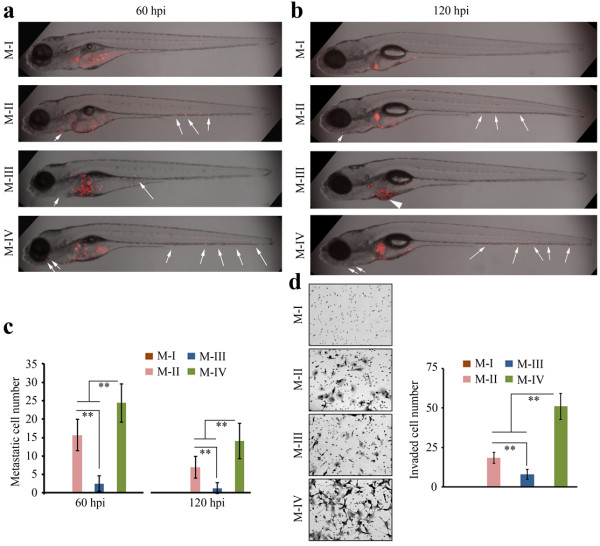
**Relative metastatic potential in human cancer cells during progression from low to high grade tumors.** When immortal, non-transformed MCF10A cells (M-I) were introduced into the zebrafish metastasis model **(a)**, no spread was seen in any of the fish analyzed at 60 hpi. The cells (M-II) recovered from low grade hyperplastic lesions in mice from MCF10AT cells (MCF10A cells transformed with activated HRAS) show increased metastatic potential (arrows). M-III cells, which were derived from well differentiated carcinomas derived from M-II cells, show reduced metastatic potential, whereas M-IV cells, recovered from aggressive adenocarcinomas derived from M-II cells, show enhanced metastatic potential. The same relationship was seen at 120 hpi **(b)** (shown as z-stack images using 40× magnification). Quantitation of metastatic cells throughout the fish body using Fiji **(c)** confirms the relationship between tumor grade and metastatic potential in fish (left). At 120 hpi, although the relative proportion of metastatic cells is seen in the M-II/M-III/M-IV comparison, the overall number of cells in each tumor grade was reduced. When the same comparison was performed using the *in vitro* invasion assay **(d)**, the relative proportion of invading cells in the different sub groups was again maintained. Data are presented as the mean of three independent experiments (n = 3) ± SEM, **P < 0.01.

To further evaluate the relationship between *in vivo* cell metastasis in zebrafish and *in vitro* cell invasion through matrigel, we studied the MCF10A system described by Tang *et al*. [27] where HRAS oncogene transformation and *in vivo* selection identified cells that have undergone progressive development into highly metastatic cells. The parental MCF10A cells (M-I), which originated from normal breast epithelial cells have been immortalized, but not transformed, and these cells do not metastasize in fish at 60 and 120 hpi (Figure 5a-b). MCF10A cells, in which the activated HRAS oncogene was expressed, are described as oncogenically initiated MCF10AT cells [28]. These cells were then passaged through mice (M-II) and can form simple ducts that can progress to benign hyperplastic lesion and occasionally carcinomas. When M-II cells were challenged in the zebrafish assay, they showed modest spread in the fish body (Figure 5a-b). Cells (M-III) that were selected from one of the carcinomas that arose from the M-II cells predominantly form well differentiated carcinomas in mice. When these cells were introduced into the zebrafish model, their low grade phenotype is reflected in the poor ability to metastasis (Figure 5a-b). During the development of these low grade tumors, however, variants arose that were more aggressive, formed carcinomas and had acquired a metastatic phenotype in mice. When these cells (M-IV) were injected into the fish model, they showed the highest level of metastasis (Figure 5a-b). We next quantified the metastatic cells in the tail region using Fiji as described above. Twelve fish per group were monitored and confocal images were taken at 60 and 120 hpi, respectively. Quantitative analysis showed that relative metastatic ability of these cells in fish was defined as M-IV > M-II > M-III > M-I (Figure 5c), which is consistent with findings from *in vitro* invasion assays (Figure 5d). Overall, therefore, in this progression model of human cancer cells, the zebrafish model accurately reports the relative level of metastatic potential in the different cells.

### Suppression of oncogenic kinases affects metastasis in zebrafish

**Figure 6:**
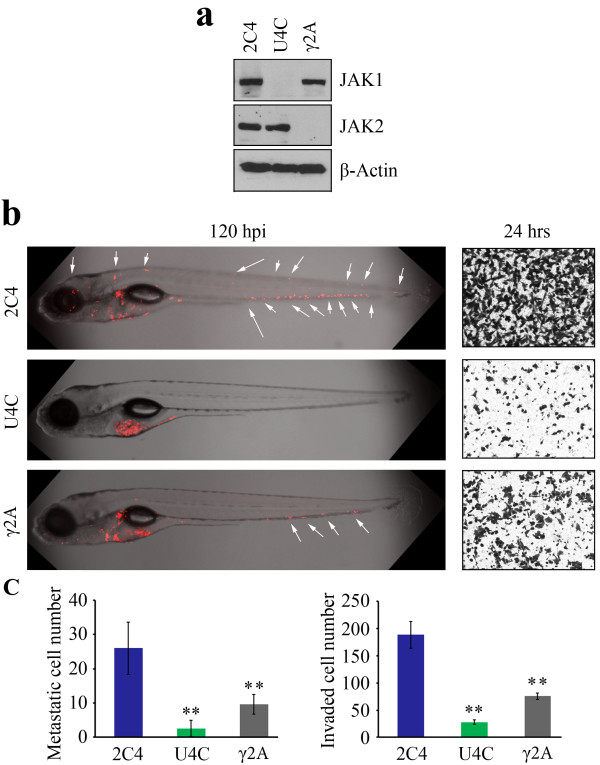
**Suppression of invasion *in vitro *leads to suppression of metastasis in zebrafish following loss of JAK1 or JAK2 expression.** Western blot analysis confirmed JAK-deficiencies in U4C and γ 2A cells **(a)**. When human 2C4 fibrosarcoma cells are injected into zebrafish (**b**, left) (shown as z-stack images using 40× magnification), extensive spread throughout the body of the fish can be seen over 120 hpi. This metastasis potential correlates with *in vitro i*nvasion (**b**, right). In both JAK1-deficient U4C cells and JAK2-deficient γ2A cells, reduced levels of metastatic spread are seen (**c**, left), which correlates with reduced invasion *in vitro* (**c**, right)*.*

One important application of a rapid and robust *in vivo* metastasis assay is the ability to quickly evaluate whether a particular genetic manipulation in cancer cells leads to loss of metastatic potential. JAK1 and JAK2, for example, have been implicated in promoting invasion and metastasis in certain cell types such as 2C4 human fibrosarcoma cells, which are highly invasive (Figure 6a). When wild-type 2C4 cells were injected into zebrafish, they show metastasis after 24 hours, which is sustained over a 5 day period (Figure 6b). The derivative U4C cells, which are genetically deficient for JAK1 [29,30], failed to spread throughout the fish body and showed virtually no invasion *in vitro* (Figure 6a). In fact, not a single cell was seen in the fish body, but sustained cell masses were seen in the perivitelline space demonstrating the persistence of viable cells in the fish over this period. In the derivative JAK2-deficient γ2A fibrosacrcoma cells [31,32], low-level metastasis was still seen but significantly suppressed compared with wild-type 2C4 cells (Figure 6b). Again, the quantitation analysis using Fiji algorithms showed the relative metastasis potential of these three different cell lines in the zebrafish reflected *in vitro* invasion assays (Figure 6c).

## Discussion

Dissection of the functional aspects of genes that impact the metastasis phenotype requires a robust assay for tumor spread. While it is accepted that, in the final analysis, rodent models should be used to evaluate metastasis, this approach is costly and inefficient as an up-front assay to determine whether a particular genetic manipulation affects the metastasis phenotype. The spontaneous metastasis assay [8,9] has shortcomings, since it cannot be used to evaluate intravasation into the blood vessels. The zebrafish model, on the other hand provides a solution to the time-consuming and costly mouse experiments, since in many cases the assay can be performed within 24–36 hours of xenotransplantation, in large cohorts of fish, providing statistical power to the results. Although we have only studied cancer cells from breast, pancreas, colon and sarcomas thus far, in all cases *in vitro* invasion ability correlated with the metastatic potential of tumor cells to spread *in vivo*. Importantly, we have shown that genetic manipulations of human cancer cells which affect invasion, also affect metastasis in fish. Although the technical dexterity needed to inject 48 hpf zebrafish can be demanding, the absence of an adaptive immune response for the first 14 days post fertilization (dpf) [33] avoids side effects and dosing issues related to using immunosuppressants [34,35]. Early studies targeting the yolk sac as a site of injection truly challenged cancer cells to enter the blood stream. The blood supply to the yolk sac is extensive since this sustains the fish for the first 5 dpf and maximizes the opportunity for intravasation. It has been shown using the *cloche* mutant fish, which do not develop a vasculature or circulation, that metastatic human cells injected into the yolk sac cannot metastasize in these fish, demonstrating the requirement for a functional circulatory system in this process [15]. Injection into the yolk sac, however, has complications apparently associated with poor resealing of the yolk sac membrane which leads to spillage of the cancer cells or yolk sac contents. The perivitelline space between the body of the fish and the yolk sac provides an alternative, which does not suffer from these associated problems. The technical challenge is successfully targeting the perivitelline space, and avoiding injection directly into the circulatory system. For this reason we examined fish after 12 hours for the presence of cells in the vasculature and excluded these fish from the analysis. Since the injection involves large numbers of fish, excluding those that have been compromised during the injection process does not have any impact on the final analysis.

Although zebrafish have been used as a model for metastasis previously, protocols between different groups were not consistent in terms of the number of cells injected, the site of injection, the age of the fish used and the method of quantitation of metastasis [18,22]. To evaluate this metastasis model more robustly, we have used a standardized protocol with a variety of different cancer cells and cell systems. In this report we clearly demonstrate that metastasis in the zebrafish correlates with *in vitro* invasion assays and in one case (DU145 cells, Figure 3) with metastasis potential in murine models of metastasis. We observed that metastatic spread in the fish was achieved as early as 24 hpi, and possibly sooner, and that the maximum tumor spread was achieved within 48 hours in most cases, without a significant increase over subsequent days. A reduction in the numbers of cancer cells, however, occurred when the analysis was extended to 5 dpi. The metastasis assay, however, if initiated at 2 dpi, can be completed before it is necessary to feed the fish (4 dpi) and, at least in the cells we tested, do not need to be followed for more that 2–3 days to evaluate metastasis. In prior studies, the number of cells injected into the fish varied between 50–2000 [14-17,19-22]. We observed that injecting too many cells can lead to mortality in our system and, following preliminary evaluation of the optimal number to demonstrate metastasis, we consistently injected cells ~300 cells per fish.

In many of our studies, the difference in the number of disseminated cells between the metastatic and non-metastatic tumors was usually striking. The same was observed in experimental cell systems where inactivation of a particular gene led to almost complete loss of invasion or metastasis, e.g. the JAK1 deficient 2C4 cells or the DU145 WASF3 knockdown cells. In these experiments, however, cells were seen outside the yolk sac region, which raised the issue of how metastasis is defined? In cell lines such as T47D, LNCAP and HT29, which are generally considered non-metastatic, our analysis showed that, even if there were disseminated cells, in the majority there were usually only between 1–5 cells in the body of the fish, where 5 cells was the exception. There are several reasons why small numbers of cells may appear in the fish body. Firstly, on rare occasions, the injection procedure could have inadvertently penetrated the vasculature and cells were introduced directly, although we screened all fish 12 hours after injection and excluded any that already showed cells outside the yolk sac region to overcome this being a major factor in the analysis. In practice this was only a very small number (<2%) for each cohort. It is also possible that cells defined as non-metastatic, are in fact weakly metastatic, and so occasional cells will disseminate into the fish. This is particularly true in experimental systems when, for example, shRNA knockdown of a particular gene is not complete, leaving some cells with gene expression levels above the threshold that will allow metastasis. It is important to note, however, that in many of our experiments involving apparently non metastatic cells, the presence of >5 disseminated cells was only seen in a minority of fish in the cohort. The main criterion for metastasis, therefore, is the presence of >5 cells in the majority of fish. In practice, however, the numbers of metastatic cells throughout the various cohorts for metastatic cells was far greater than 5 as shown in Figure 4 (35–55 cells after 48 hours). The number of disseminated cells becomes particularly important when defining relative metastatic potential, as seen in the MCF10A continuum. In this case the overall number of cells found outside the yolk sac area correlated with the invasiveness of the cells in vitro. We expect, however, most metastasis assays will want to determine whether ablation or overexpression of a gene leads to changes in metastatic potential, and following the protocol described here will facilitate this determination.

Although we were limited by the number of clinical samples available to us, the demonstration that primary human cancer cells can survive, grow, and metastasize in zebrafish provides a very encouraging proof-of-principle and opens opportunities to evaluate the metastatic potential in primary cells from biopsies or following surgery, which can have important advantages for clinical management of the patient. Even though it may take 2–3 weeks to establish the primary cell cultures, the presence of metastatic cells in a tumor for which there is a well-differentiated histopathology, could affect the future screening and management protocol. In addition, the well- established systems [36-38] for drug screening in zebrafish, opens up the possibility of identifying therapeutics that can target metastasis on a tumor-by-tumor basis, so providing a personalized approach to individual tumors.

## Conclusions

In summary, we provide a side-by-side critical evaluation showing that zebrafish can evaluate the metastatic potential of human cancer cell lines and primary tumors. The ability to perform these analyses in large cohorts of fish allow for robust statistical analysis in a short time frame (24–120 hours) and provides a rapid means of evaluating whether genetic manipulation of cells, or cell origin, affects metastasis in vivo. Where it has been investigated, the metastasis phenotype in fish is identical to that in rodents with the advantages that individual cells can be imaged during the metastasis process.

## Methods

### Cell culture and primary lung cancer cell isolation

Human breast cancer cells (MDA231 and T47D), prostate cancer cells (DU145 and LNCaP), pancreatic ductal adenocarcinoma cells (AsPC-1 and BxPC3) and colon cancer cells (SW620 and HT29) were obtained from the American Type Culture Collection (ATCC, Rockville, MD, USA). The parental human fibrosarcoma cell line 2C4, the JAK1-deficient U4C and JAK2-deficient γ2A derivatives were a gift from Dr. Ivo P. Touw (Erasmus University Medical Center, The Netherlands) and were cultured in DMEM supplemented with 10% (v/v) fetal bovine serum (FBS). The normal MCF10A breast epithelial cell line (M-I) and its derivatives M-II, M-III and M-IV [27,39], were a gift from Dr. Shuang Huang (Georgia Regents University, USA). The M-III and M-IV cells were maintained in DMEM/F-12 supplemented with 5% horse serum. The culture medium for the M-I and M-II was similar but also included 20 ng/ml recombinant human EGF (R&D Systems, Minneapolis, MN, USA), 0.5 μg/ml hydrocortisone (Sigma-Aldrich, St. Louis, MO, USA), 10 μg/ml insulin (Sigma-Aldrich) and 100 ng/ml cholera toxin (Sigma-Aldrich). Primary lung cancer cells were obtained immediately after resection, disaggregated with trypsin and cultured in DMEM for 5–10 days.

### *In vitro* invasion assay and experimental metastasis mouse model

To measure cell invasion potential, matrigel invasion assays were performed as described previously [7,25] using Transwells (BD biosciences, San Diego, CA, USA) with 8-μm pore size filters. The invading cells were fixed in 3.7% paraformaldehyde and stained with 0.5% crystal violet in 2% ethanol. The lower surface of the filter was photographed and the invading cells were counted from six fields at 200× magnification. Each experiment was performed in triplicate on at least three occasions. For experimental metastasis, 6-week-old male SCID mice were injected with 1 × 10^6^ WASF3 knockdown DU145 cells or the knockdown control cells through the lateral tail vein [9]. Mice were sacrificed 3 months after injection and the lung tissues were processed for hematoxylin and eosin (HE) staining. All experimental procedures were approved by the Animal Care and Use Committee of Georgia Regents University.

### Cell preparation and transplantation

For cell labeling, cells were incubated with cell tracker CM-Dil (Invitrogen, Carlsbad, CA, USA) at a final concentration of 2.5 μg/ml for 4 min at 37°C followed by 15 min at 4°C. To remove unincorporated dye, cells were rinsed twice with phosphate-buffered saline (PBS), and then resuspended at a higher concentration (5 × 10^6^ cells/ml). Before injection, CM-Dil labeled cells were assessed for viability using trypan blue exclusion and only samples in which there was >90% viability were used. We also evaluated the cells for uniform red staining and membrane integrity using a Zeiss Axiovert microscope (Zeiss, Thornwood, NY, USA) before being transplanted into the fish.

### Zebrafish husbandry and the metastasis model

Zebrafish were maintained using established temperature and light cycle conditions as previously described [40,41]. All experimental procedures were approved by the Animal Care and Use Committee of Georgia Regents University. The *Tg(kdrl:EGFP)* transgenic fish line was a gift from Dr. Daniel Wagner (Department of Biochemistry and Cell Biology, Rice University, USA). For zebrafish xenotrasplantation, 48 hpf wild-type AB or *Tg(kdrl:EGFP)* strains of transgenic zebrafish embryos were dechorionated and anaesthetized in 0.3× Danieau's solution containing phenythiourea (PTU, Sigma-Aldrich) and 0.04 mg/ml tricaine (Sigma-Aldrich) before human cell injection. Approximately 300 CM-Dil labeled human cells were injected into the perivitelline cavity of each embryo, and zebrafish were maintained in 0.3× Danieau's solution containing PTU for 1 h at 28°C. After confirmation of a visible cell mass at the injection site, zebrafish were transferred to an incubator and maintained at 34°C.

### Confocal imaging

Living zebrafish embryos were anesthetized using 0.04 mg/ml tricaine and were then embedded in a lateral orientation in 0.5% agarose. Serial sections were captured using an Olympus FLUOVIEW™ FV1000 laser scanning confocal microscope (Olympus, Tokyo, Japan) and 2.5 μm z-step intervals. Low magnification (×4 objective) was used to provide an overview of the tumor cell metastasis pattern throughout the fish, and higher magnification (×20 objective) was used to define the precise localization of metastatic cells and foci within the zebrafish. Z-stack images were processed using ImageJ/Fiji as previously described [40,42].

### Automated cell counting and statistical analysis

The custom Fiji (ImageJ2) software package [26] was used for automated cell counting. Briefly, a 190–255 intensity threshold was set to select cells and the ‘analyze’ particle tool was used with default selection of cell size and cell shape during counting. A Fiji macro was generated using the ‘record’ function to streamline analyses and remove bias. To count the metastatic cell number in the tail, the fish tails were selected for cell counts using the polygon selection tool. A *P*-value of 0.05 or less was considered to be statistically significant and determined by the Student’s *t*-test. Values of three or more experiments were given as mean ± SEM.

## Competing interests

The authors declare no competing financial interests.

## Authors’ contributions

YT and JC designed the experiments; YT performed the molecular and cellular experiments; YT, XX and DW performed the fish husbandry and imaging, YT and SW performed the image analysis; JM provided input into zebrafish experiments; YT, JC and JM wrote the manuscript. All authors read and approved the final manuscript.

## Pre-publication history

The pre-publication history for this paper can be accessed here:

http://www.biomedcentral.com/1471-2407/13/453/prepub
